# An ultra-fast method for designing holographic phase shifting surfaces

**DOI:** 10.1038/s41598-023-43815-2

**Published:** 2023-10-02

**Authors:** Akash Biswas, Constantinos L. Zekios, Stavros V. Georgakopoulos

**Affiliations:** Department of Electrical and Computer Engineering, Florida International University, Miami, FL 33174 USA

**Keywords:** Engineering, Electrical and electronic engineering

## Abstract

Holographic phase-shifting surfaces (PSSs) have been proven to offer a cost-effective solution for enabling passive arrays to mechanically steer their beams toward desired directions. However, even though the principle of operation of PSSs is straightforward, designing a PSS is very challenging, because it involves an extremely high computational time, which in turn limits their usage and development. Notably, traditional design approaches of PSSs, with *N* number of layers that have *M* different variations of conductive patches, need $M^N$ full-wave simulations to be properly characterized. To address these challenges that are associated with the design of PSSs and reduce the needed computational effort, we present here a semi-numerical approach that enables the efficient design of holographic PSSs. Specifically, by representing an *N*-layer PSS unit-cell as *N* cascaded networks, where each network represents one layer of the PSS that has *M* different designs of sub-wavelength resonators, we only need to conduct $N \times M$ full-wave simulations to collect all the required data needed to analyze the performance of the PSS. In turn, by utilizing the multiplication property of ABCD parameters we can evaluate very efficiently all the $M^N$ different combinations that characterize our PSS. To validate the accuracy of our design methodology, a 1-D beam steerable antenna system is designed that is comprised of a circularly polarized holographic metasurface antenna (HMA) and a hybrid PSS, both operating at 30 GHz. Comparisons between our semi-numerical results, full-wave simulations, and measurements demonstrate an angular error of less than $0.7^{\circ }$.

## Introduction

Next-generation wireless networks, such as 5G, 5G-beyond, and 6G, are expected to transform our way of life. In fact, 5G and 6G networks will connect any device with any other one, any person with anybody, any business with any service, any computer with any network, offering connectivity at any place anywhere, and anytime with any context^1^. Towards this end, significant advancements have been made to develop novel communication systems and devices that can support the needs of next-generation wireless networks^2^. Also, the bandwidth of devices and systems needs to be expanded to cover the millimeter wave (mmWave) and THz frequency regime. However, to move to higher frequencies, major technical challenges must be addressed so that communication systems provide robust, accurate, effective, and reliable communication links^3^. Specifically, cost-effective, compact, high-efficiency, and high-gain antennas with multi-beam and dynamic beamsteering capabilities are urgently needed to support the development of 5G and 6G systems that can provide (a) real-time multi-user-to-multi-machine or multi-machine-to-multi-machine operation, (b) high traffic capacity (10 Mbps/m$^2$), (c) high data rates (20 Gbps for downlink and 10 Gbps for uplink), (d) uniform user experience of 50 Mbps, and (e) latency of 1 ms^4^.

In the literature, several different solutions have been proposed to address these requirements, such as phased arrays (PAs), high-gain transmitarrays (TAs) and reflectarrays (RAs), metasurface antennas (MAs), and leaky wave antennas (LWAs). Notably, each of these antennas has its own advantages and disadvantages. For example, PAs can dynamically steer their beams in any desired direction with a high level of beam agility^5^. However, PAs have complex designs with power-hungry beamforming mechanisms, which significantly increase their cost and their profile. TAs, RAs, and MAs are simple cost-effective solutions that achieve very high gains due to their large apertures, but they have limited beamsteering capabilities compared to PAs. Finally, LWAs can steer their beams at any desired angle, but they need to sweep their frequency to achieve this, and they are limited to scanning in one principal plane.

A different solution is the use of mechanically steerable antennas. Mechanically steerable antennas have been extensively used since 1935, with the introduction of the first radar system, and are suitable for applications where beam resolutions in the order of 1 mrad and response times in the order of 100 ms are required^6^. Notably, mechanical beam-steering methods include Risley prism-based phase shifting surfaces^7^, two-axis gimbaled scanners^8^, lenslet arrays^9^, and micro-electromechanical systems (MEMS)^10^. In this article, we focus on phase-shifting surfaces (PSS).

Risley prism-based phase shifting surfaces have been extensively used in both optics and microwave frequencies and particularly in the fields of satellite communications, laser communications, infrared countermeasure, space observation, biomedicine, machining, and imaging^11–18^. Their principle of operation is very simple. By changing the angle of rotation of two properly designed prisms, an incident beam can be steered towards a desired direction^19^. For example, in Ref.^20^, a pair of rotating stepped-dielectric phase transformers were designed to demonstrate a Ka-band beam-scanning antenna system for a conical angular sector of $40^{\circ }$. In Ref.^21^, a similar approach was followed to steer the beam of a rectangular horn antenna with a maximum apex angle of $104^{\circ }$. Notably, even though, these stepped-dielectric phase transformers are simple to design and they can be easily scaled to higher frequencies, they are bulky, and have a very high profile, thereby limiting their use to very few applications where the aperture size or the height of the radiating element is not important. To address this drawback, Gagnon et al.^22^, introduced, more than a decade ago, an electrically thin phase-shifting surface using holographic-based principles. Numerous designs have been developed since then. For example, in Ref.^23^, Afzal et al., used a pair of properly designed metasurfaces to steer the beam of a resonant cavity antenna in a conical region with an apex angle of $102^{\circ }$. In Ref.^24^, a similar approach was used for the case of a radial-line slot array demonstrating a maximum elevation angle of $40.6^{\circ }$, while in Ref.^25^, the same principle was utilized for the case of an all-metal beamsteering antenna. Finally, in Ref.^26^, quasi-nondiffractive beam generation was demonstrated proving the applicability of PSSs in near-field applications, such as short-range ultra high-speed communications, imaging, and wireless energy transmission.

In summary, significant research has been conducted on metasurface-based (or holographic-based) PSSs; however, past efforts have mainly focused on creating PSSs that fulfill specific design requirements, such as high gain, low insertion loss, wide phase variation, etc. Notably, even though the principle of operation of PSSs is straightforward (e.g. Refs.^22, 27–29^), designing a PSS is very challenging because it involves an extremely high computational time, which in turn limits the usage and development of PSSs. Specifically, metasurface-based PSSs are multilayer structures with several sub-wavelength metallic resonators. It is the design of these resonators that requires thousands or even millions of full-wave simulations, which is computationally very expensive. To alleviate this problem, we introduce here a semi-numerical ultra-fast design approach for designing metasurface-based PSSs. Our approach uses the multiplication property of ABCD matrices of cascaded networks. Namely, by decomposing an *N*-layer PSS into *N* distinct models, we only need to perform $N \times {M}$ full-wave simulations (where *M* is the number of different designs of conductive patches for each layer), whereas traditional approaches for studying PSSs require a number of operations in the order of $M^N$. In Refs.^30, 31^, we showed preliminary simulation results of our approach. Here, we extend our initial work by: (a) providing a complete design methodology for the analysis of a metasurface-based PSS with different types of resonators, (b) conducting a complete error analysis by using full-wave simulations as a reference for our error calculations, and (c) demonstrating a complete beamsteerable antenna prototype with measured and simulated results that are in excellent agreement to each other. Similar approaches using ABCD matrices can be found in the literature, particularly in the design and analysis of metasurfaces. Notable examples include the work of Pfeiffer et al.^32^ and Chen et al.^33^, who employed stacked multi-layer unit-cells and impedance sheet conditions to achieve optimal field transformations in metasurfaces. One may also see Budhu et al.^34^, for a review on stacked multi-layer impedance sheet realization of bianisotropic unit cells which utilizes the ABCD approach. Additionally, Olk et al.^35^, and Kelly et al.^36^ have further advanced these approaches by incorporating near-field coupling effects and offering comprehensive design methodologies for electromagnetic Huygens’ metasurfaces.

In what follows, in “Theory” section, we present our proposed semi-numerical design methodology. In “Methods” section, we conduct a complete error analysis of the proposed design methodology, and we validate our methodology by designing, and fabricating a 1-D beam-steerable antenna. Finally, “Discussions” section concludes our work.

**Figure 1:**
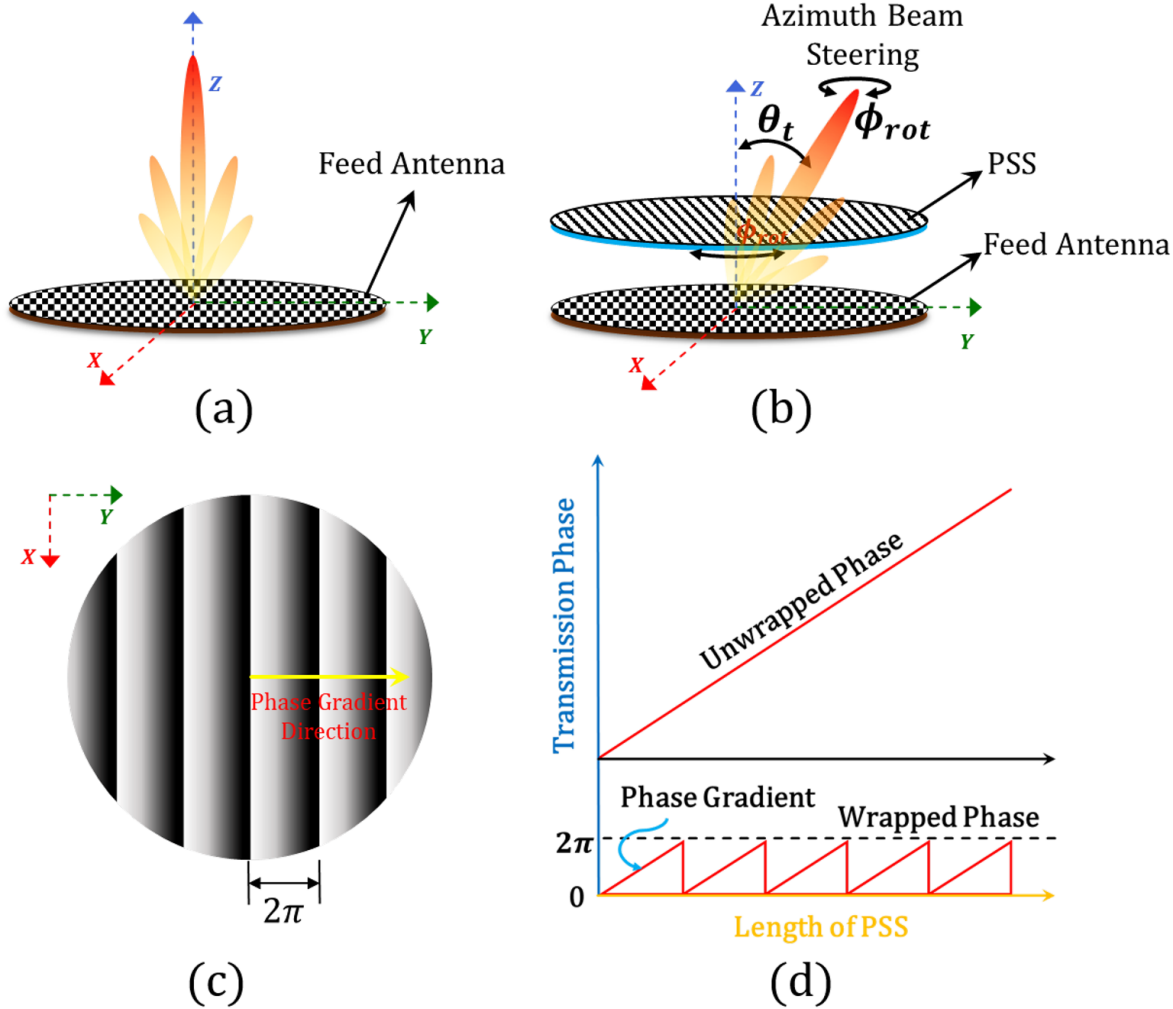
Schematic diagram of a 1-D beam-steerable antenna system consisting of a metasurface feed antenna and a metasurface-based phase shifting surface. (**a**) Feed antenna. (**b**) 1-D beam-steerable antenna system. (**c**) Schematic of a PSS phase distribution. (**d**) Transmission phase gradient of PSS.

## Theory

### Conventional PSS design methodology

A traditional PSS-based beam-steerable antenna is comprised of the following: (a) a base or feed high gain antenna (e.g., a horn antenna, an array, a metasurface, etc.), and (b) a passive phase shifting surface (e.g., a pair of dielectrics wedges, a set of metasurface-based surfaces, etc.). Figure 1 shows a schematic view of a 1-D beam-steerable antenna, that is comprised of a metasurface feed antenna (see Fig. 1a) and a metasurface-based PSS placed above the feed antenna (see Fig. 1b). Notably, the feed antenna produces a fixed broadside beam. Also, the PSS is designed as a multilayered phase-gradient band-pass surface (see Fig. 1c) that consists of properly transmissive ($S_{12} <-2$ dB) engineered unit-cells of metallic resonators with a $360^{\circ }$ phase coverage (see Fig. 1d). By properly choosing the phase gradient of the PSS, the broadside beam of the feed antenna is tilted towards a desired $\theta _t$ angle. Notably, the relation between beam tilt angle and the phase gradient of the PSS is established as^24^:

1 $$\begin{aligned} \phi _p = \left( \frac{2\pi }{\lambda _0}\right) S sin(\theta _t), \end{aligned}$$

where *S* is the periodicity of the unit-cell, $\lambda _0$ is the free space wavelength at the operating frequency, and $\phi _p$ is the progressive phase shift between two adjacent unit-cells. Therefore, the entire design process of the PSS depends on the unit-cell properties. These unit-cell properties are thoroughly chosen to manipulate electromagnetic beams based on the principles of microwave holography (e.g., Refs.^27, 37–39^). Specifically, they record the interference pattern between a reference beam, often a spherical/planner wavefront, and a desired beam, usually a plane wave. Therefore, the PSS comprised of an array of unit-cells functions as a recorded hologram. When illuminated with a reference wave from a source antenna, it enables complex beam steering (e.g., Refs.^22, 28^) or beam-splitting operations (e.g., Ref.^40^), regenerating the desired wavefront. In particular, as it has already been shown in the literature, a traditional holographic-based unit-cell design is comprised of multiple layers of dielectrics and resonant metallic patches (see Fig. S1a in the Supplementary Material) properly chosen to cover the required $360^{\circ }$ transmission phase range that tilts the beam towards a desired $\theta _t$ angle. In general, the higher the number of layers is, the higher the phase range we can achieve. Notably, by varying the sizes of the different metallic patches, in the unit-cell, different phase shifts are observed. Also, the unit-cell size should be kept in sub-wavelength dimensions to avoid generating grating lobes, while its overall thickness should be kept as low as possible aiming for low-profile and compact designs. Finally, the chosen unit-cell and, therefore, its resonant patches should be rotationally symmetric to enable the seamless steering of the feed antenna’s broadside beam when the PSS rotates in the azimuthal plane.

To build a PSS, following the conventional design approach, a metallic resonator is chosen (e.g., a square patch), and a multi-layer unit-cell structure, comprised of *N* layers of this resonator, is modeled. Traditionally, full-wave simulations are conducted, where a plane wave excites the unit-cell, and periodic boundary conditions are appropriately applied (see Fig. S1 in the Supplementary Material), to obtain the transmission amplitude and phase shift responses of this unit-cell. By changing the geometrical properties of the unit-cell (e.g., dielectric thicknesses, and sizes of the metallic patches) different transmission amplitudes and phase responses are obtained. Usually, the thickness of the unit-cell is preset based on fabrication limitations or application constraints; and, therefore, only the size of the metallic patches can vary. Therefore, to investigate the electromagnetic performance of an *N*-layer unit-cell with *M* different designs of conductive patches for each layer, $M^N$ full-wave simulations are required. Figure S3 in the Supplementary Material shows the amplitude and phase responses in terms of transmission coefficient for a 2-, 3- and 4-layer unit-cell based on square metallic patches. Importantly, it demonstrates that despite the extensive data collection (resulting in a continuum of values), none of these cases can achieve the full 360-degree transmission phase range required. Thus, even though, the principle of operation of PSSs is straightforward, properly designing a PSS is extremely challenging, due to the very high computational time and the large number of full-wave simulations that are needed. This problem becomes even more severe in cases where we want to combine different resonators in a single PSS design (this type of PSS is usually called hybrid), because it exponentially increases the number of full-wave simulations needed to characterize our unit-cells. Specifically, these hybrid unit-cells can consist of different resonators at each layer including, (a) single printed circuits, such as rectangular patches, with a complexity level *M* equivalent to the single-element unit-cell design, (b) single printed circuits with a complexity level exceeding *M*, for example, a Jerusalem cross (e.g., Ref.^41^) compared to a rectangular patch, or (c) multiple printed circuits with a complexity level exceeding *M*, such as a split-ring resonator (e.g., Ref.^42^) compared to a rectangular patch. Using different resonators in a single PSS is a common approach when designing metasurface-based PSSs, as we will discuss later in “Methods” section, because it significantly improves transmission amplitude and phase responses compared to traditional PSSs that use one type of resonator.

**Figure 2:**
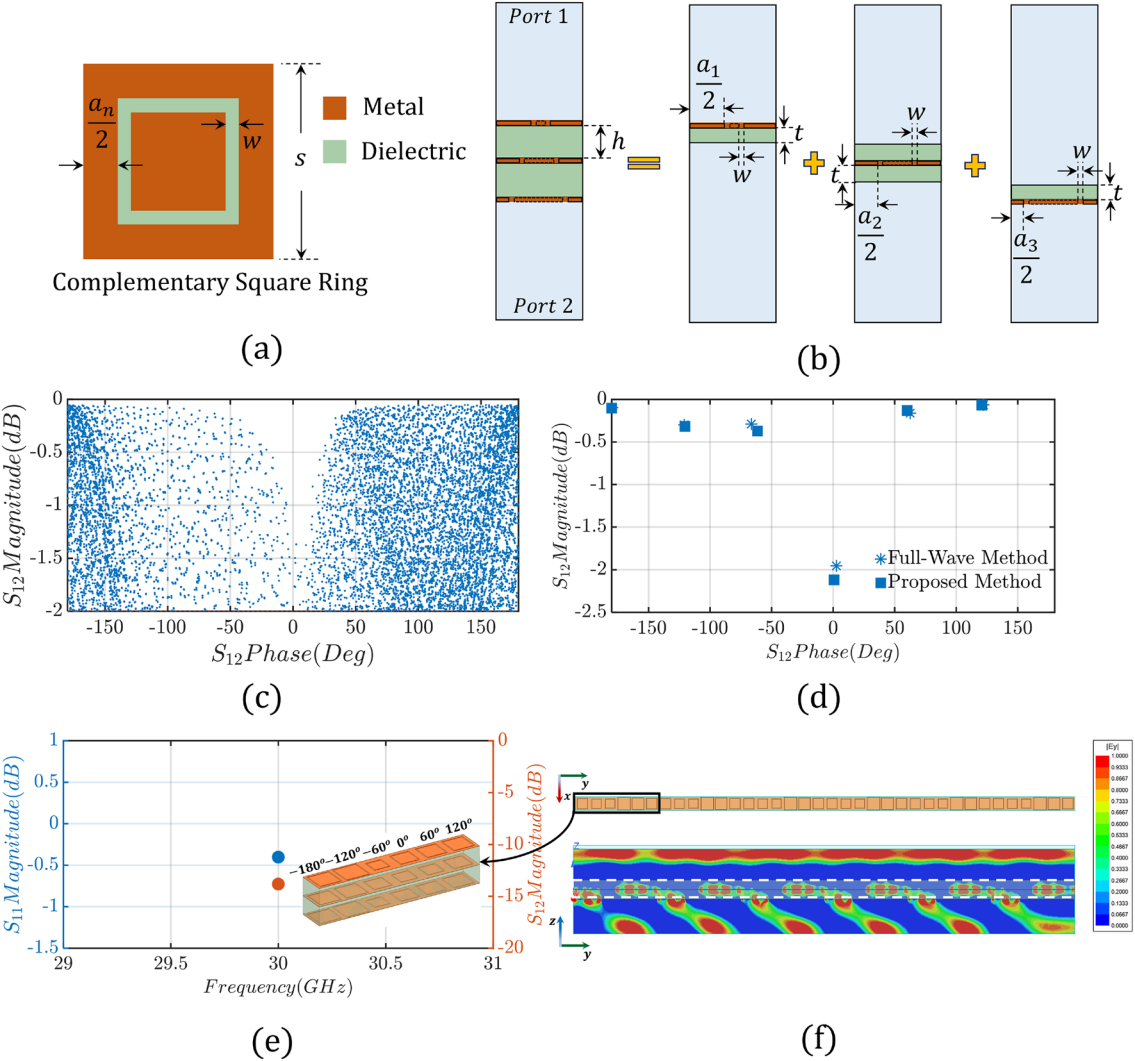
Design schematic view of our proposed methodology for the case of a three-layer unit-cell comprised of complementary square ring resonators. (**a**) Complementary square ring (CSR) unit-cell ($S=3$ mm, $a_n\le 3$ mm, $w=0.1$ mm). (**b**) A three-layer PSS unit-cell is split into three separate unit-cells (sections) to enable the development of our proposed design method ($h=2$ mm, and $t=1$ mm for the case of $t_{Total} = 2 h = 4$ mm). (**c**) Transmission phase and amplitude lookup table (database) of CSR unit-cell. (**d**) Comparison of the transmission amplitude and phase, between proposed (semi-numerical) and conventional (full-wave) methodology, for the case of $n=6$ unit-cells that offer the required $\phi _p=60^{\circ }$ phase progression. (**e**) Transmission and reflection coefficient of the CSR super-unit-cell. (**f**) E-field ($|E_y|$) cross-section distribution of an infinite 1-*D* CSR PSS.

### Proposed semi-numerical methodology

To significantly reduce the computational burden of analyzing holographic (or metasurface-based) PSSs, we propose a semi-numerical ultra-fast design approach that uses the multiplication property of *ABCD* matrices of cascaded networks. Specifically, by expressing an *N*-layer PSS unit-cell as a network of *N* distinct models, we only need to perform $N \times {M}$ full-wave simulations (where *M* is the number of different designs of conductive patches for each layer). In turn, we can evaluate all the $M^N$ possible designs by using the *ABCD* multiplication property. In what follows, the steps of our proposed methodology are presented in detail:

- *Step 1* Choose an *N*-layer unit-cell design of total thickness $t_{Total}$ and divide it into *N* distinct models. Each model consists of a metallic resonator covered on top and/or bottom by the dielectric material of thickness $t=t_{Total}/2(N-1)$ (see Fig. 2).
- *Step 2* Choose one (or more) metallic resonators (e.g., rectangular patch, circular patch, slotted patch, etc.) and define *M* different variations that solely depend on its (or their) geometrical properties (size^25^, the azimuthal orientation of the resonator^7, 43^, shape^44^, etc.).
- *Step 3* Conduct $N\times {M}$ simulations and get the corresponding S-parameters. Namely, model each layer separately using all the different *M* variations of the corresponding resonator in an infinite array simulation setup (e.g., the design under study is excited by a plane wave from both its top and bottom faces, and is surrounded by periodic boundary conditions). Notably, the relative position of the $N{\text {th}}$ layer, with respect to the entire unit-cell, should not change (see Fig. 2b), and the corresponding S-parameters should be evaluated right above and below (at the top and bottom faces, respectively, as shown in Fig. S7 in the Supplementary Material) the corresponding layer. This is very important to accurately evaluate the phase of the corresponding S-parameters.
- *Step 4* Convert the S-parameters into their corresponding ABCD matrices, and use the multiplication property of ABCD matrices of cascaded networks:
  2 $$\begin{aligned} ABCD_{total} = [ABCD_{1}, ABCD_{2},{\cdots }ABCD_{M}]_1\bigotimes [ABCD_{1}, ABCD_{2},{\cdots }ABCD_{M}]_2\bigotimes \nonumber \\ {\cdots }\bigotimes [ABCD_{1}, ABCD_{2},....ABCD_{M}]_N, \end{aligned}$$
  where $\otimes$ is used to denote the Kronecker product between the *M* *ABCD* matrices of each layer. By performing the ABCD matrix multiplication a lookup table (a database) of all the $M^N$ possible designs is created.
- *Step 5* Define the desired $\theta _t$ angle for steering the broadside beam of the feed antenna and find the required progressive phase $\phi _p$ using 1. Based on the $\phi _p$ angle, evaluate the required number of unit-cells (*n*) needed to achieve the $360^{\circ }$ phase gradient on the PSS. The number of unit-cells is calculated as $n=2\pi /\phi _p$.
- *Step 6* Choose *n* appropriate unit-cells that (a) satisfy the phase progression $\phi _p$ requirement when they are arranged co-linearly (see inset of Fig. 2e), and (b) achieve low reflection coefficient $S_{11}$ (e.g., typical below $-\,10$ dB) and a transmission coefficient $S_{12}$ of typically above $-\,2$ dB. If they do not satisfy the complete $360^{\circ }$ phase range, go back to step 2 and choose a different resonator type and/or increase the number of layers.
- *Step 7* Create a super-unit-cell that consists of the *n* unit-cells defined in the previous step and conduct a full-wave infinite array simulation to evaluate its electromagnetic characteristics (see Fig. 2e,f). If both the reflection and transmission coefficients of the entire super-unit-cell satisfy the desired requirements proceed to the next step, otherwise go back to step 6 and choose a different combination of unit-cells.
- *Step 8* Create the final PSS design by arranging periodically the super-unit-cell from Step 7 (see Fig. S2 in the Supplementary Material) and conduct full-wave simulations to obtain the desired electromagnetic performance.

In the next section, we investigate the accuracy of our proposed design methodology and we design a 1-D beam-steerable antenna system using both the traditional and the proposed approach.

## Methods

In this section, we conduct an error analysis of our semi-numerical methodology, and in turn, we validate it with the design of a 1-D beam-steerable antenna system.

### Error analysis

To validate our design approach a thorough study is conducted by designing holographic PSSs utilizing traditional metallic resonators (e.g., complementary square ring, square patch, square ring), and we compare our results with the results observed when the conventional design methodology (see “Conventional PSS design methodology” section) is employed.

We start our analysis with the three-layer PSS unit-cell of Fig. 2a that consists of three complementary square ring (CSR) resonators. Notably, the frequency of operation is chosen at 30 GHz. Also, we arbitrarily choose to design a PSS that steers a broadside beam excited by a feed antenna at angle $\theta _t=33.7^{\circ }$. Based on this $\theta _t$ value, using (1), the phase progression, $\phi _p$, is equal to $60^{\circ }$. For this $\phi _p$ value, to achieve a $360^{\circ }$ phase range coverage, $n=6$ unit-cells need to be used. Following the sub-wavelength principle of operation of metasurfaces, the unit-cell periodicity is chosen at $S=3$ mm (this is $\lambda _0/3.33$ at 30 GHz). Also, the width *w* of the slot, etched in the complementary square ring (see Fig. 2a), is kept constant at 0.1 mm for this analysis. Finally, two Duroid/Rogers 5880 ($\epsilon _r=2.2$ and $tan\delta =0.0009$) substrates are used with a height of $h=2$ mm (this height is equal to $\lambda _0/5$ at 30 GHz) each. For this example, the sizes of the complementary rings change by varying $a_n$ from 0.2 to 2.5 mm with an increment of 0.025 mm ($M=93$ variations for each layer). Notably, the total number of different combinations of rings for this example is equal to 804,357 (that is $M^N$ = $93^3$), therefore, 804,357 full-wave simulations need to be run with the conventional approach to fully characterize the performance of this PSS. These results are used as reference data to evaluate the accuracy of our approach. For our method, as explained above, only $3 \times 93 = 279$ full-wave simulations are needed. Notably, this number can be even reduced to $2 \times 93 = 186$ full-wave simulations since the top and bottom layers, in this example, are identical. Therefore, the electromagnetic properties of the bottom (top) layer, for example, can be obtained by simply inverting the ABCD matrix of the top (bottom) layer. Then, we use the multiplication property of *ABCD* parameters for cascaded networks to calculate the performance of all the 804,357 different designs for this PSS. Figure 2c shows all the amplitude and phases evaluated for insertion loss less than $-\,2$ dB (each point corresponds to a different unit-cell with different combinations of CSRs and different sizes). Data with insertion loss greater than $-\,2$ dB are omitted since they have no practical significance as discussed above. Notably, this CSR unit-cell covers the entire $360^{\circ }$ phase range, and we can identify different unit-cell combinations that satisfy the desired $\phi _p=60^{\circ }$ phase progression (see in Fig. 2c, for example, the areas at $\pm \,180^{\circ }$, $\pm \,120^{\circ }$, $\pm \,60^{\circ }$, and $0^{\circ }$). The accuracy of our approach is determined by comparing the results of our method to the full-wave simulation reference data. Figure 2d, indicatively, compares the amplitude and phase responses of our method to the ones observed by the conventional approach, for the $n=6$ unit-cells that offer the required $60^{\circ }$ phase progression computed above. Using (3) and (4):

3 $$\begin{aligned} Error_{phase}= \left| \frac{\phi _{ref}-\phi }{2\pi }\right| \times 100\%, \end{aligned}$$

4 $$\begin{aligned} Error_{amplitude}= \left| T_{ref}-T\right| \times 100\%. \end{aligned}$$

We evaluate the percentage of the absolute error between the two approaches, showing a maximum amplitude and phase error of 1.5% and 1.4%, respectively at $h = 0.2\lambda$.

**Figure 3:**
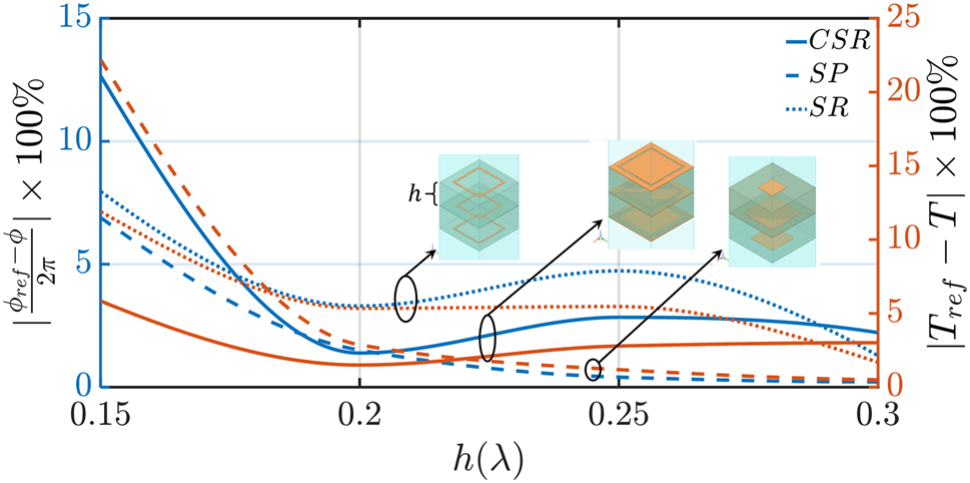
Phase and amplitude percentage error as the total height of a three-layer PSS unit-cell varies. Three different unit-cell designs are shown here (CSR, SP, and SR) which have been commonly used in the literature. Here, $\phi _{ref}$ and $T_{ref}$ correspond to phase and amplitude responses based on full-wave simulations, and $\phi$ and *T* represent phase and amplitude responses obtained from our proposed method.

A similar analysis is conducted for both the square patch (SP) and square ring (SR)-based unit-cells, showing a maximum error of 2.9% and 5.3%, respectively at $h = 0.2\lambda$. Figure S4a–d of our Supplementary Material present the equivalent analyses showing the corresponding responses for both SP and SR unit-cells. To further investigate the accuracy of our proposed methodology, we study the phase and amplitude percentage errors as the total height of a three-layer PSS unit-cell varies for all the three different unit-cell designs modeled here (CSR, SP, and SR). Figure 3 shows the corresponding results where the worst-case scenario (e.g., the maximum error) among all cell variations has been used. To evaluate the percentage errors we use as reference phase, $\phi _{ref}$, and reference amplitude, $T_{ref}$, the phase and amplitude responses of the full-wave simulations. Notably, $\phi$ and *T* represent the phase and amplitude responses obtained by our proposed method. The blue line corresponds to the phase percentage error and the red line corresponds to the amplitude percentage error. As we can see from these results, the square ring introduces a higher overall error compared to the other two resonators, while the square patch exhibits minimal error at $h = 0.2\lambda$, which is our point of interest here. Nevertheless, what is important to note here, is that for all three resonators, both the phase and amplitude percentage errors increase significantly for heights smaller than $0.175\lambda$. This behavior is expected and is attributed to the strong mutual coupling between the resonant patches, when they are brought close to each other. Even though this seems to limit the applicability of our proposed methodology to designs that are thicker than $\lambda /6$, in reality, the majority of antenna designs that operate at millimeter wave frequencies have thicknesses that are greater than $\lambda /5$. Therefore, our proposed method should be applicable to most practical designs. Also, Olk et al.^35^ recently introduced a method that takes into account the mutual coupling between the different layers of a unit-cell design, similar to our ABCD approach. Thus, by utilizing Olk’s approach we expect to enhance the accuracy of our proposed methodology, leading to a significant reduction in both amplitude and phase errors. However, such an analysis is not conducted in this study and is left as a future work.

**Figure 4:**
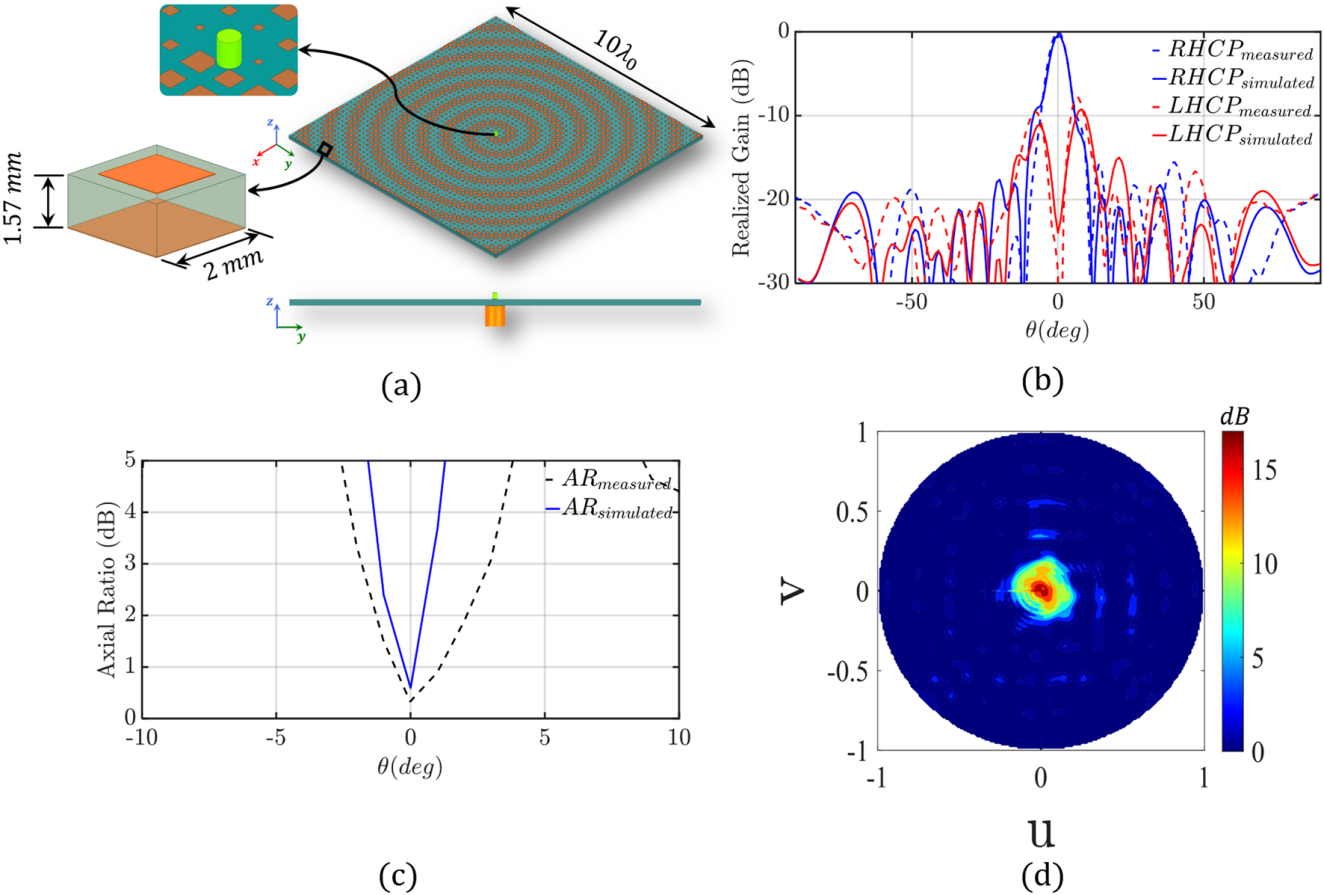
(**a**) Isometric and side view of our designed holographic metasurface antenna (HMA). In the inset, the monopole feed antenna, and the HMA unit-cell are shown. Comparison of simulated and measured responses; (**b**) 2-*D* normalized radiation pattern of the HMA at $\phi =0^{\circ }$ cut-plane, and (**c**) axial ratio. (**d**) Simulated 3-*D* radiation pattern contour plot.

**Figure 5:**
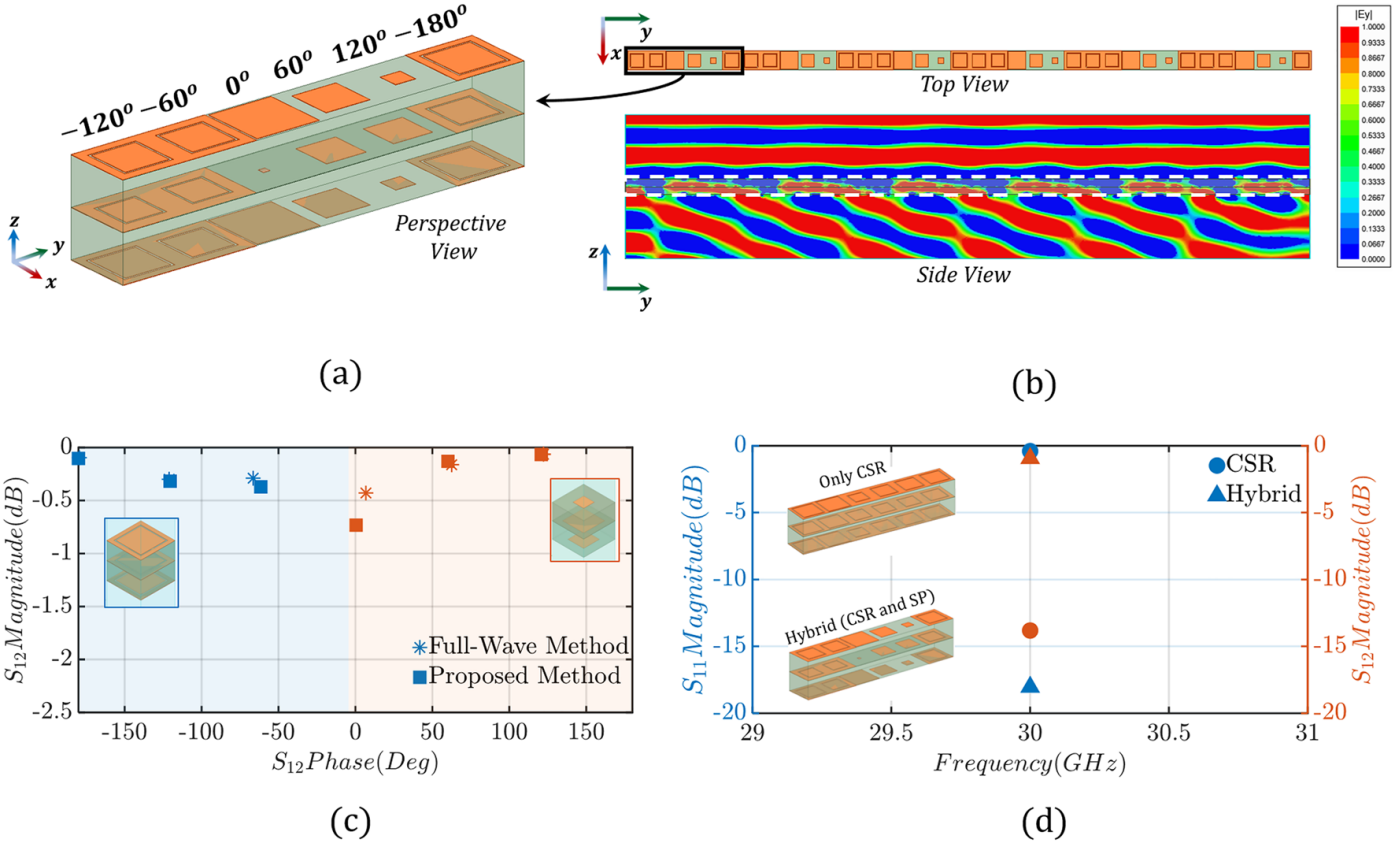
(**a**) Hybrid PSS super-unit-cell. (**b**) E-field ($|E_y|$) cross-section distribution of an infinite 1-*D* hybrid PSS. (**c**) Comparison of the transmission amplitude and phase, between proposed (semi-numerical) and conventional (full-wave) methodology, for the case of properly chosen $n=3+3$ CSR and SP unit-cells, that when are combined offer the required $\phi _p=60^{\circ }$ phase progression. The geometrical dimensions for the data points of these unit-cells are tabulated in Table ST1 in the Supplementary Material. (**d**) Transmission and reflection coefficient of the CSR only and hybrid super-unit-cells.

### Validation: design of a 1-D beam-steerable antenna

To validate the accuracy of our proposed methodology, we design a $10\lambda _0 \times 10\lambda _0$ 1-D beam-steerable antenna system that operates at $f=30$ GHz consisting of a right-hand circularly polarized (RHCP) HMA with a broadside beam, and a properly designed holographic PSS that steers the broadside beam at $\theta _t=33.7^{\circ }$. Notably, our proposed method of designing PSS does not limit the use of a base antenna to HMA only, and other antenna types (e.g., antenna arrays, radial-line slot arrays, etc.) can be used. In what follows, we, briefly present the design of the HMA, and then, we design our PSS and simulate and measure the entire antenna system.

#### Holographic metasurface antenna (HMA)

A $10 \lambda _0 \times 10\lambda _0$ square spiral holographic metasurface antenna (HMA) is properly designed to feed our 1-D beam-steerable antenna system. Figure 4a shows our HMA which consists of properly designed square metallic patches over the ground plane, and a monopole antenna that excites the HMA at its center. The design process of the HMA is available in Ref.^45^, and is omitted here for reasons of brevity. A Duroid/Rogers 5880 substrate is used with a thickness of 1.57 mm, a relative permittivity of 2.2, and a loss tangent of 0.0009. Figure S6 in the Supplementary Material shows our prototype. Notably, a 2.4 mm connector is used as our monopole feed. Figure 4b indicates excellent matching between simulation and measured normalized gain. The HMA achieves a maximum broadside gain of 18.3 dBi. Also, as we can see from Fig. 4b,c, the HMA is right-hand circularly polarized (RHCP) with an axial ratio close to 1 dB at the broadside direction for both simulated and measured responses. Figure 4d shows the simulated $3-D$ radiation pattern contour plot.

**Figure 6:**
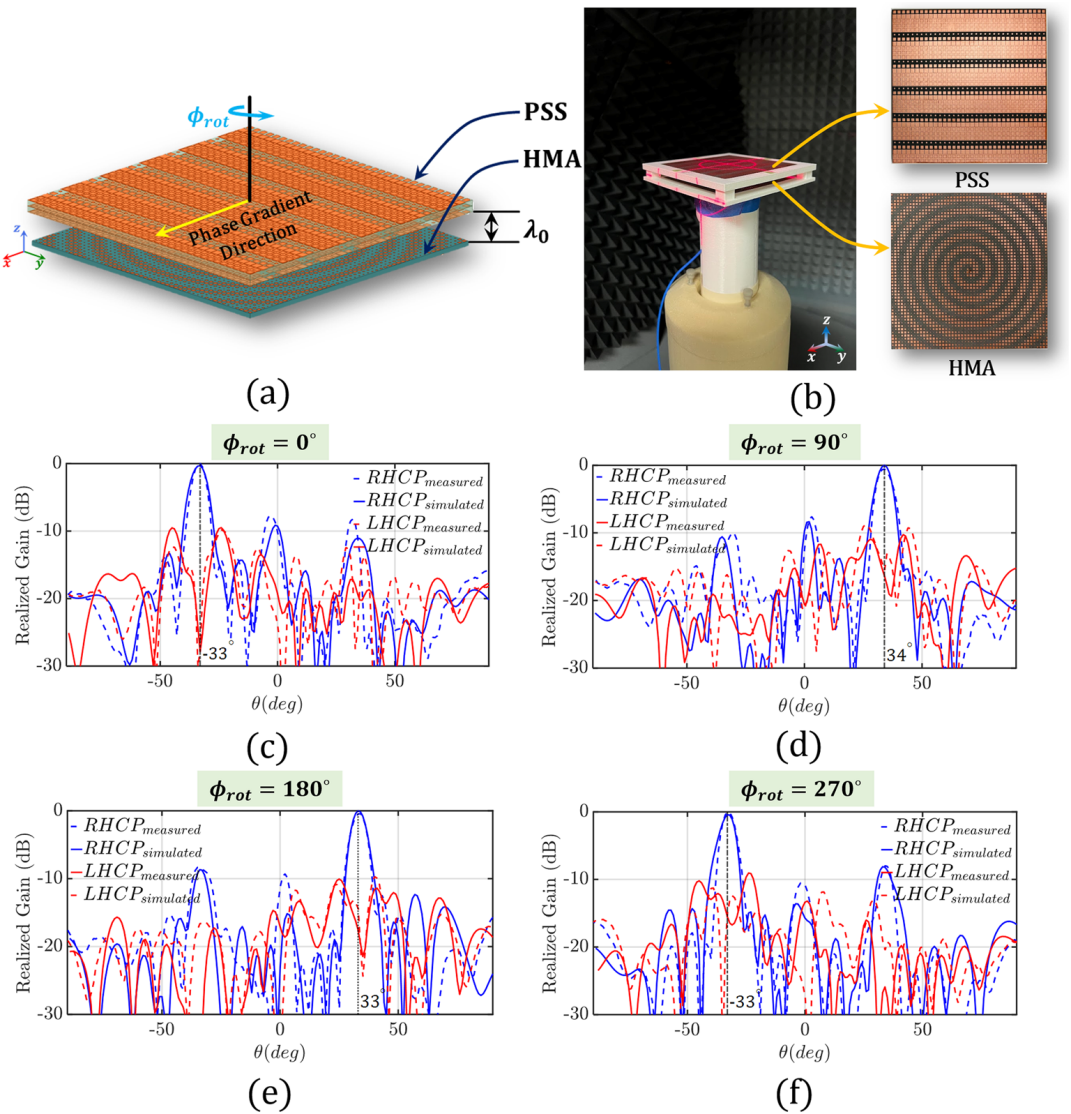
(**a**) Isometric view of the 1-D beam-steerable antenna system with the PSS placed above the HMA in a distance $d=\lambda _0$. (**b**) Fabricated prototype of 1-D beam-steerable antenna system with measurement setup. The PSS (top) and HMA (bottom) are shown in the inset. 2-*D* normalized co-pol and cross-pol radiation pattern at different $\phi _{rot}$ rotation angles of (**c**) $0^{\circ }$, (**d**) $90^{\circ }$, (**e**) $180^{\circ }$, and (**f**) $270^{\circ }$, respectively.

#### Beam-steerable antenna system

As discussed earlier, our goal is to design a 1-D beam-steerable antenna system that operates at $f=30$ GHz and points its beam at $\theta _t=33.7^{\circ }$. To achieve this goal, we need to properly design our PSS. First, we start by choosing the appropriate unit-cell design that can (a) cover the entire $360^{\circ }$ phase range, and (b) achieve a transmission coefficient above $-\,2$ dB, as discussed above. Notably, in “Theory” section we investigated the design of three different PSSs based on three different three-layer unit-cell designs (CSR, SP, and SR). Figure 2c and Fig. S4a,c show the corresponding amplitude and phase responses, where, it is seen that only the CSR-based unit-cell designs satisfy the two above-mentioned requirements of $360^{\circ }$ phase range and transmission coefficient of $S_{12}$ >$-2$ dB. The next step is to choose $n=6$ unit-cell designs (we proved this in “Theory” section) to steer the broadside beam at $33.7^{\circ }$. Figure 2d shows the amplitude and phase for a set of candidate unit-cell designs for our CSR resonator. Next, following Step 7 of our design methodology, we create a super-unit-cell comprised of the 6 CSR unit-cells of Fig. 2d, and we conduct full-wave infinite array simulations to evaluate its electromagnetic performance. Figure 2e,f show the corresponding responses. As we can see from these responses, even though the field distribution is directed towards the expected direction of $33.7^{\circ }$, the CSR-based super-unit-cell has high reflection and low transmission. This is despite the fact that all the individual unit-cells have very good transmission coefficients, and it is attributed to the mutual coupling between the different unit-cells that has not been taken into account when we conduct infinite array full-wave simulations of the corresponding unit-cells. Notably, this is not a drawback of our methodology, but it is a common behavior that also appears in the conventional approach, where also infinite array simulations are conducted for characterizing the corresponding unit-cells. Only very recently a methodology was introduced in Ref.^46^, where the transverse coupling between unit-cells has been considered. Nevertheless, to address this challenge, hybrid PSSs have been introduced that use combinations of different unit-cells. Therefore, moving forward with our design, we create a super-unit-cell that is comprised of both CSR and SP unit-cell designs. Figure 5a shows this design. Notably, as we can see from Fig. 5c, we use 3 CSR unit-cell designs to cover the $(-180^{\circ },-60^{\circ })$ phase range, and 3 SP unit-cell designs to cover the $(0^{\circ },120^{\circ })$ phase range. To properly select the unit-cell designs that we need to retain and replace, an intermediate electromagnetic analysis is required to examine the mutual coupling between the different unit-cells. In this analysis, we construct our super-unit-cell by progressively adding different unit-cells and monitoring the resulting transmission and reflection coefficients. When undesirable transmission or reflection coefficients occur, signifying high mutual coupling, we replace the corresponding unit-cell design with one that meets our desired criteria. In our specific case, after conducting this analysis, we identified the 4th unit-cell design within the super-unit-cell shown in Fig. 2e (inset) as responsible for the excessive reflection. Notably, although the 3rd and 5th unit-cell designs in Fig. 2e (inset) did not introduce significant reflection, we replaced them to maintain symmetry in the super-unit-cell. Figure 5b shows the field distribution of the hybrid PSS that is directed towards the expected direction of $33.7^{\circ }$. Moreover, Fig. 5d shows the response of the hybrid super-unit-cell where both its reflection and transmission coefficients are at $-\,18.04$ dB and $-\,0.94$ dB, respectively. These responses are acceptable, and, therefore, we use this as our final super-unit-cell based on which we build our PSS.

Notably, our PSS has a total aperture of $100 \times 100$ mm$^2$, which corresponds to $10\lambda _0\times 10\lambda _0$ at the operating frequency of 30 GHz, and a total thickness of 4 mm, consisting of two dielectric and three metallic layers. To model and build the PSS, we use Duroid/Rogers 5880 as the substrate with a relative permittivity of 2.2 and a loss tangent of 0.0009. Due to the unavailability of a 2 mm custom thickness dielectric substrate in the market, to fabricate our prototype, we combined two single-sided copper laminate 1.57 mm and 0.38 mm Duroid/Rogers 5880 substrates with a prepreg (Rogers 6700 of 0.038 mm thickness) to construct a 2 mm dielectric substrate. Finally, two of these 2 mm layers were joined with the prepreg (Rogers 6700 of 0.038 mm thickness). A detailed view of the PSS and its layers is provided in Fig. S5 of our Supplementary Material. Next, we place our hybrid PSS (see Fig. S2b in our Supplementary Material) above the HMA we designed in “Holographic metasurface antenna (HMA)” section in a distance of $\lambda _0$ (10 mm at 30 *GHz*) and characterize the entire beam-steerable antenna system. To hold our PSS in place, we used a 3D-printed plastic mold. Figure 6a shows an isometric view of the entire antenna system and Fig. 6b shows the fabricated prototype placed inside our MVG MicroLab anechoic chamber^47^. In the inset of Fig. 6b, the fabricated PSS and HMA are shown, respectively.

**Figure 7:**
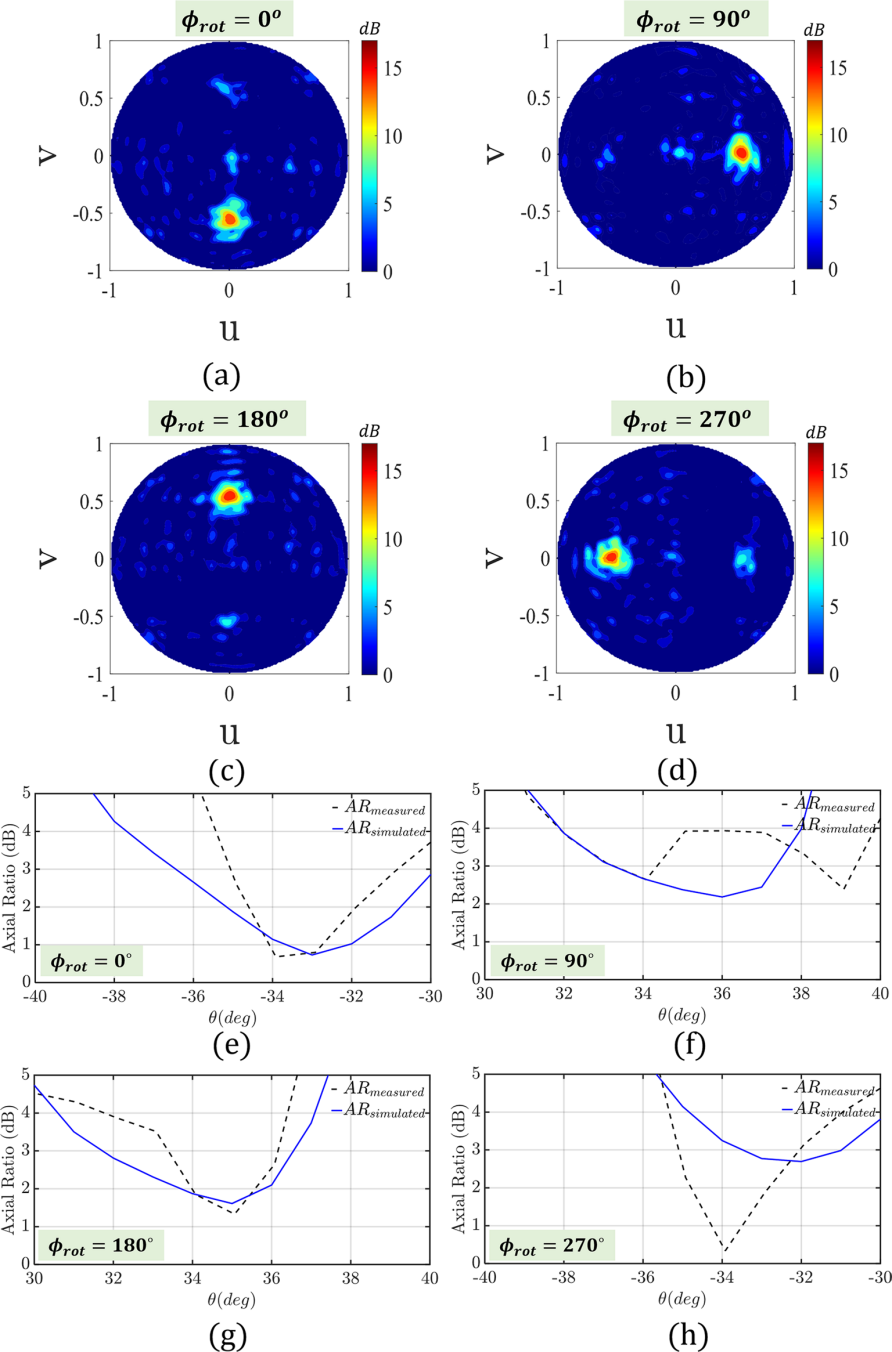
(**a–d**) Simulated 3-*D* radiation pattern contour plot, and (**e–h**) simulated and measured axial ratio (AR) for $0^{\circ }$, $90^{\circ }$, $180^{\circ }$, and $270^{\circ }$ rotation angles ($\phi _{rot}$), respectively.

To examine the beam-steering capability of our hybrid PSS, we conducted a series of tests. Namely, we rotated the PSS at four different azimuthal angles ($\phi _{rot}=0^{\circ }$, $90^{\circ }$, $180^{\circ }$, and $270^{\circ }$), while keeping the HMA at the same position. Figure 6c–f compare the simulated with the measured radiation patterns. Our results clearly demonstrate that the PSS preserves the RHCP radiation pattern of the HMA, while tilting the beam to $\theta _t=33^{\circ }$. Only a $0.3^{\circ }{-}0.7^{\circ }$ beam angle error is observed, which is of no practical significance. Table 1 tabulates the beam direction for all four cases as well as the corresponding realized gains for both simulated and measured responses. As we can see from this table, a very stable gain of $16 \pm 0.4$ dBi is achieved. Notably, this gain is 2.7 to 1.9 dB lower compared to the 18.3 dBi gain of the HMA when no PSS is used. This gain reduction is totally expected and is attributed to (a) the insertion loss of 0.94 dB introduced by the PSS (see Fig. 5d), and (b) the cosine roll-off as we steer the beam from broadside to $33.7^{\circ }$. The slight asymmetry we observe with respect to the different rotation angles is attributed to the fact that the HMA is not entirely symmetric in the azimuthal plane. Also, the measured gains in some cases are slightly higher compared to the simulated ones. This is not surprising and it is attributed to the limitations of our computational resources to evaluate with high accuracy the entire structure. Figure 7a–d present the contour plots of the corresponding $3-D$ radiation patterns for all the rotation angles. Finally, Fig. 7e–h show, the axial ratio of our design, which is always maintained below 3 dB for all the $\phi _{rot}$ rotation cases at $\theta =33^{\circ }\pm 0.7^{\circ }$.

**Table 1:** Antenna system realized gain and axial ratio for different PSS rotation angles ($\phi _{rot}$).

$\phi _{rot}$	Beam direction $\theta$ in $[0, \pi ]$		Realized gain (dBi)		AR (dB)	
	Simulated	Measured	Simulated	Measured	Simulated	Measured
$0^{\circ }$	${33}^o$	${33}^o$	15.6	15.89	0.74	0.79
$90^{\circ }$	${34}^o$	${34}^o$	16.2	15.57	2.67	2.66
$180^{\circ }$	${33}^o$	${34}^o$	16.4	15.82	2.31	1.85
$270^{\circ }$	${33}^o$	${33}^o$	16.1	16.34	2.77	1.87

## Discussions

A semi-numerical computationally efficient method for the design of holographic phase-shifting surfaces was presented. Specifically, by representing N-layer PSS unit-cells as N-cascaded networks, where each layer can have *M* different designs of patches, we showed that only $N \times M$ full-wave simulations are needed, instead of $M^N$ full-wave simulations (that traditional design approaches require) to completely characterize the EM performance of our PSS. Notably, by utilizing the multiplication property of ABCD parameters, we proved that all the $M^N$ evaluations can be very effectively evaluated through the $N \times M$ data we generated with our method with a total percentage error of less than 3% for both amplitude and phase for heights as small as $\lambda /5$. To validate the accuracy of our design methodology, a 1-D beam steerable antenna system comprised of a circularly polarized HMA and a hybrid PSS was designed at 30 GHz. Comparisons between our semi-numerical results, full-wave simulations, and measurements demonstrated an angular error of less than $0.7^{\circ }$.

### Supplementary Information

Supplementary Information.

## Data Availability

All data generated or analyzed during this study are included in this published article [and its Supplementary Information files].
