# Supplementary material for: An ultra-fast method for designing holographic phase shifting surfaces
Source: Sci Rep. 2023 Oct 2;13:16511. doi: 10.1038/s41598-023-43815-2 (PMC10545707; doi:10.1038/s41598-023-43815-2)
Supplement: Supplementary file 1 — Supplementary Information. [file 41598_2023_43815_MOESM1_ESM.pdf]

Supplementary Material
(An Ultra-Fast Method for Designing Holographic Phase Shifting Surfaces)

Akash Biswas[1,+], Constantinos L. Zekios[1,*], and Stavros V. Georgakopoulos[1]

[1]Department of Electrical and Computer Engineering, Florida International University, Miami, FL 33174, USA,
[+]abisw002@fiu.edu
[*]kzekios@fiu.edu

1 Analysis of a Square Patch (SP) Unit-cell

As discussed in Section 2.1 of our manuscript, to properly design a metasurface-based PSS that tilts the broadside beam of a feed antenna towards a specific θ_t angle, the unit-cells of the PSS need to be designed to achieve a progressive phase shift ϕ_p:

$$\phi_p = \left(\frac{2\pi}{\lambda_0}\right) Ssin(\theta_t) \tag{1}$$

where S is the periodicity of the unit cell, λ_0 is the free-space wavelength at the operating frequency and ϕ_p is the progressive phase shift between two adjacent unit cells. For example, to design a PSS that operates at 30 GHz, we choose a periodicity of 3 mm that is less than one-third of the free-space wavelength at the operating frequency. To tilt the broadside beam of the feed antenna at 33.7°, a progressive phase shift of $\phi_p = 60^\circ$ is calculated. Figure S3 shows the amplitude and phase responses in terms of transmission coefficient for a 2-, 3- and 4-layer unit-cell design based on square metallic patches. Here, a transmission coefficient of less than $-2\ dB$ is chosen as an acceptable value, and, therefore, only the corresponding responses are shown. As we can see, from these responses, the higher the number of layers is, the higher the phase range we can achieve. However, as we increase the number of layers in our unit cell, the number of full-wave simulations rapidly increases. For example, for the results we show here, we conducted 226, 339, and 452 full-wave simulations assuming $M = 113$ patch variations for the 2-, 3- and 4-layer unit cell design, respectively, that correspond to $7m\ 44s$, $12m\ 22s$, and $17m\ 33s$ simulation times, respectively, in a CPU server of 32-core Intel Xeon processor with 700 GB of RAM.

Table ST 1. Geometrical Dimensions of the Hybrid PSS (CSR and SP unit cells)

Transmission Phase	Geometrical Dimensions (in mm)			Proposed Method		Full-Wave Simulation	
	Complementary Square Ring (CSR)			Transmission Phase (Deg)	Transmission Amplitude (dB)	Transmission Phase (Deg)	Transmission Amplitude (dB)
	a_1	a_2	a_3				
-180°	0.7	1	0.7	-179.99	-0.10	-179.42	-0.10
-120°	0.825	1.15	0.75	-120.42	-0.32	-121.07	-0.30
-60°	0.875	1.175	0.925	-61.47	-0.37	-66.46	-0.29
	Square Patch (SP)						
	l_1	l_2	l_3				
0°	2.8	0.3	2.825	0.45	-0.73	5.87	-0.47
60°	1.975	1.775	1.975	60.80	-0.03	59.57	-0.04
120°	0.9	1.75	0.9	119.84	-0.02	120.05	-0.02

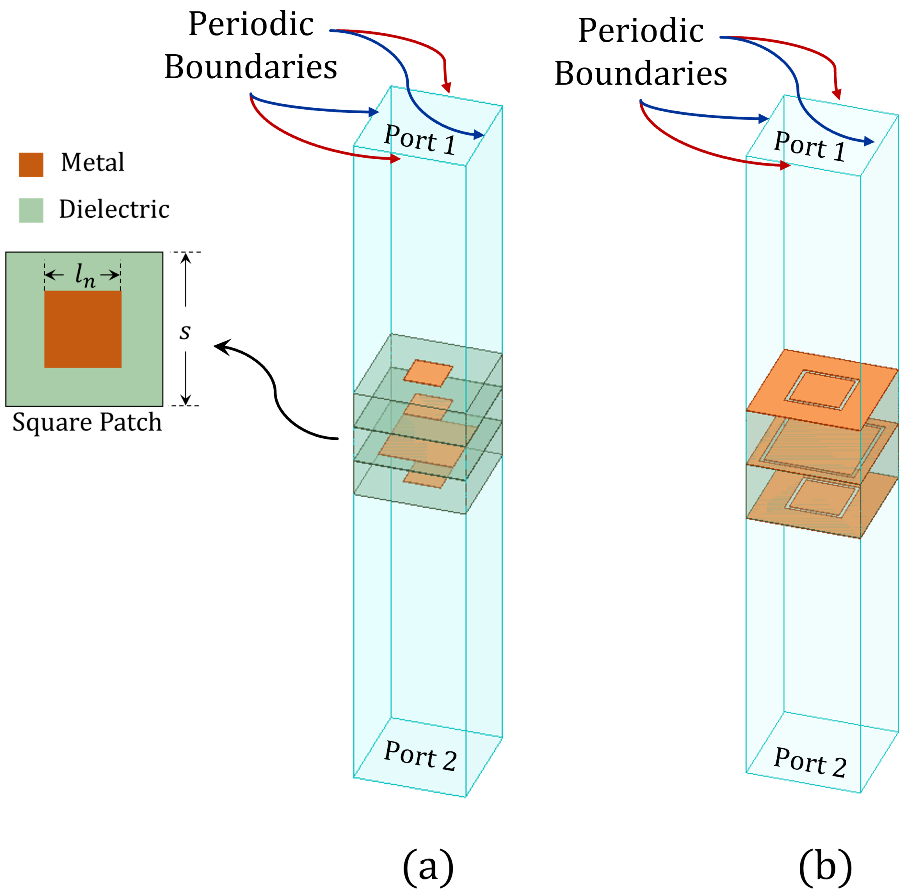

Figure S 1. (a) A 4-layer square patch (SP) unit-cell ($S = 3\ mm$, $l_n \leq 3\ mm$). (b) A 3-layer complementary square ring (CSR) unit-cell.

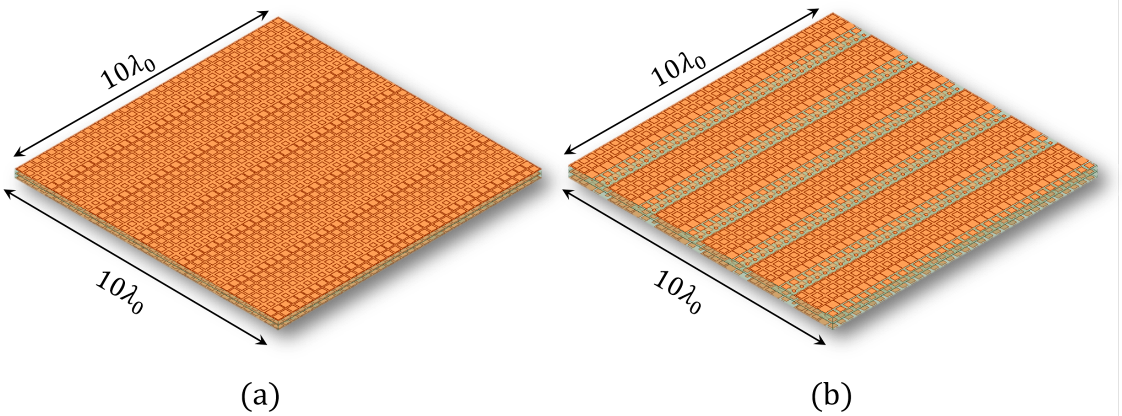

Figure S 2. A complete $10\lambda_0$ PSS built with (a) only CSR unit-cell, and (b) hybrid unit-cell.

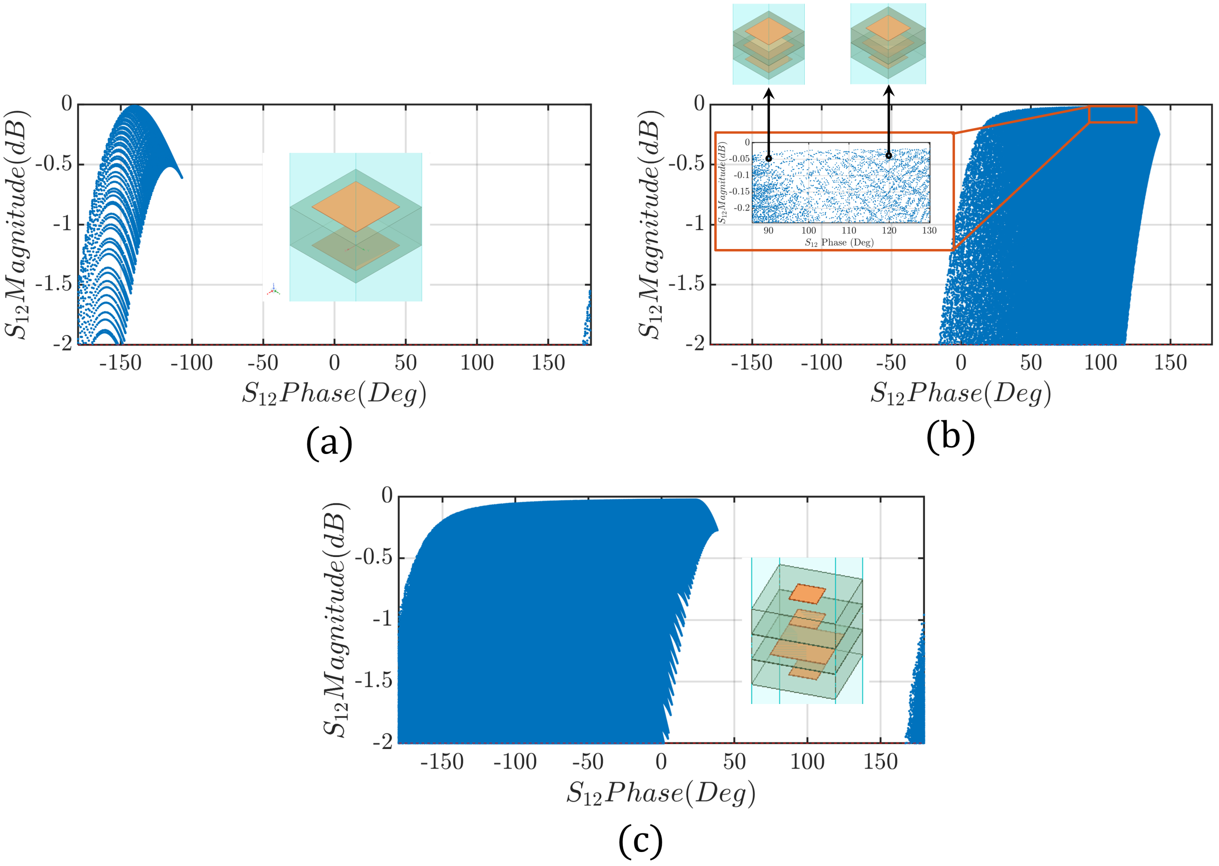

Figure S 3. Transmission phase and amplitude lookup table (database) of (a) 2-layer, (b) 3-layer (a zoomed view of the data points is shown in the inset), and (c) 4-layer of square patch (SP) unit-cell, respectively. Here, the unit-cell dimensions ($S = 3$ *mm*) and substrate thicknesses ($h = 2$ *mm*) are kept the same for all three cases. Notably, this Figure demonstrates that despite the extensive data collection (resulting in a continuum of values), none of the three cases can achieve the full 360-degree transmission phase range required.

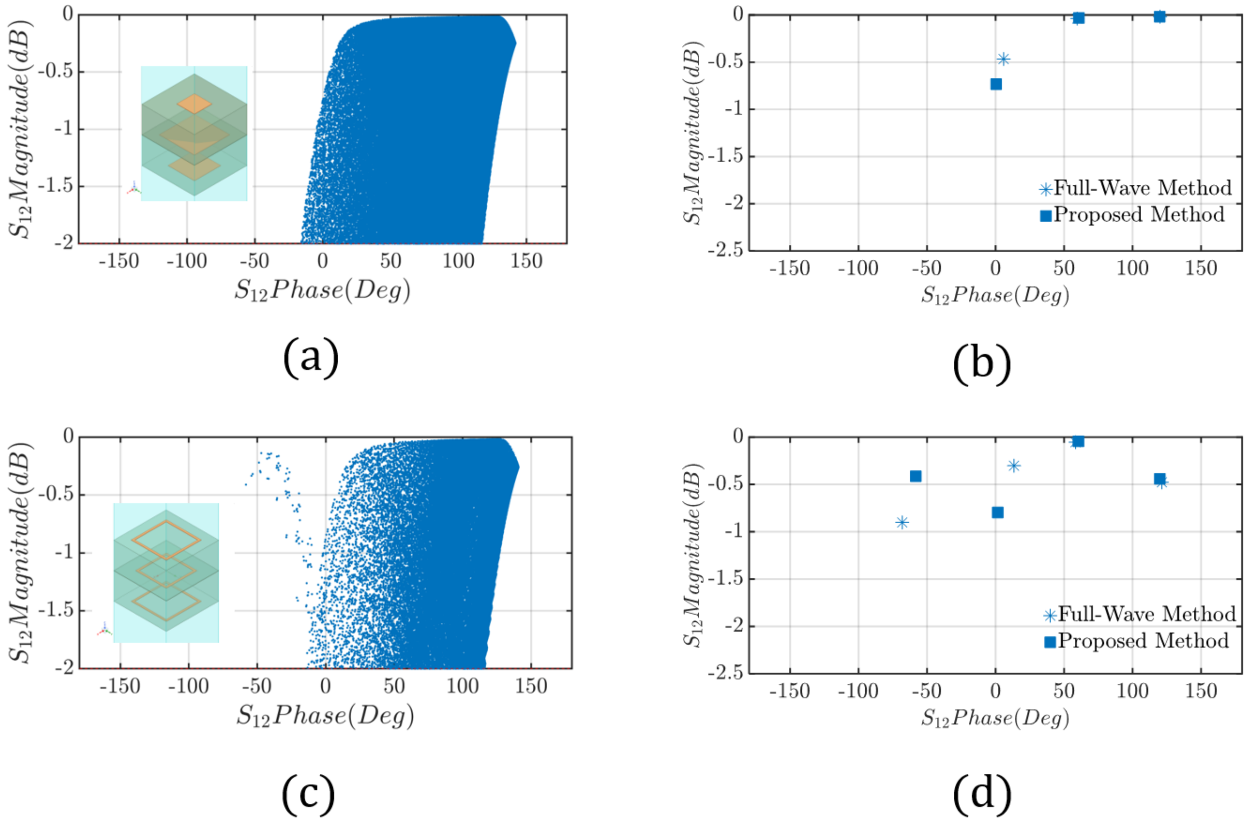

Figure S 4. (a) Transmission phase and amplitude lookup table (database) of a three-layer SP unit-cell. (b) Comparison of the transmission amplitude and phase, between proposed (semi-numerical) and conventional (full-wave) methodology, for the case of 3 unit-cells that offer the required $\phi_p = 60°$ phase progression. (c) Transmission phase and amplitude lookup table (database) of a three-layer SR unit-cell. (b) Comparison of the transmission amplitude and phase, between proposed (semi-numerical) and conventional (full-wave) methodology, for the case of 4 unit-cells that offer the required $\phi_p = 60°$ phase progression. Here, all the unit-cell dimensions ($S = 3\ mm$) and substrate thicknesses ($h = 2\ mm$) are kept the same for all cases.

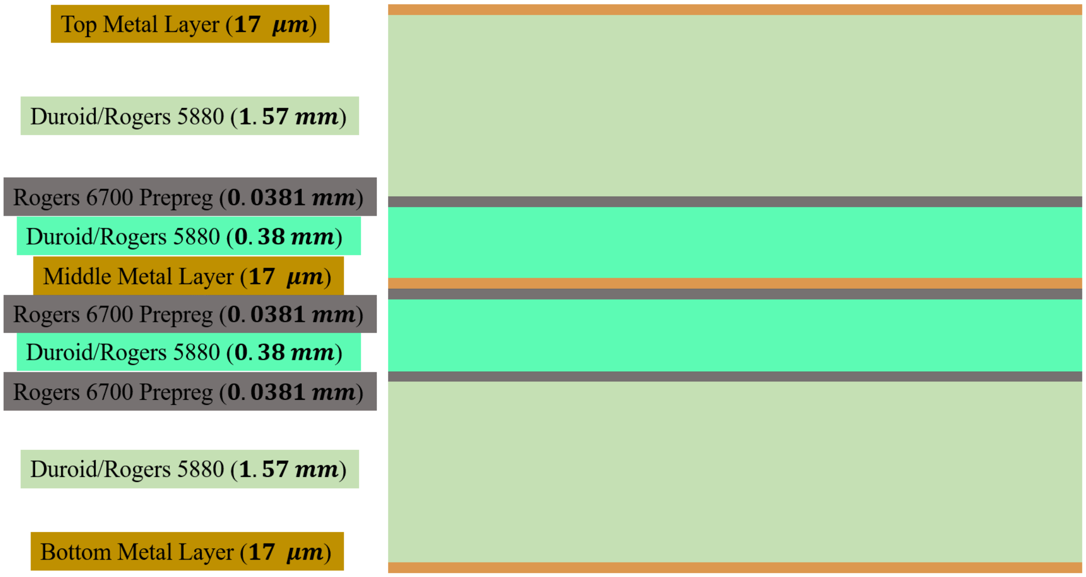

Figure S 5. PSS stack-up.

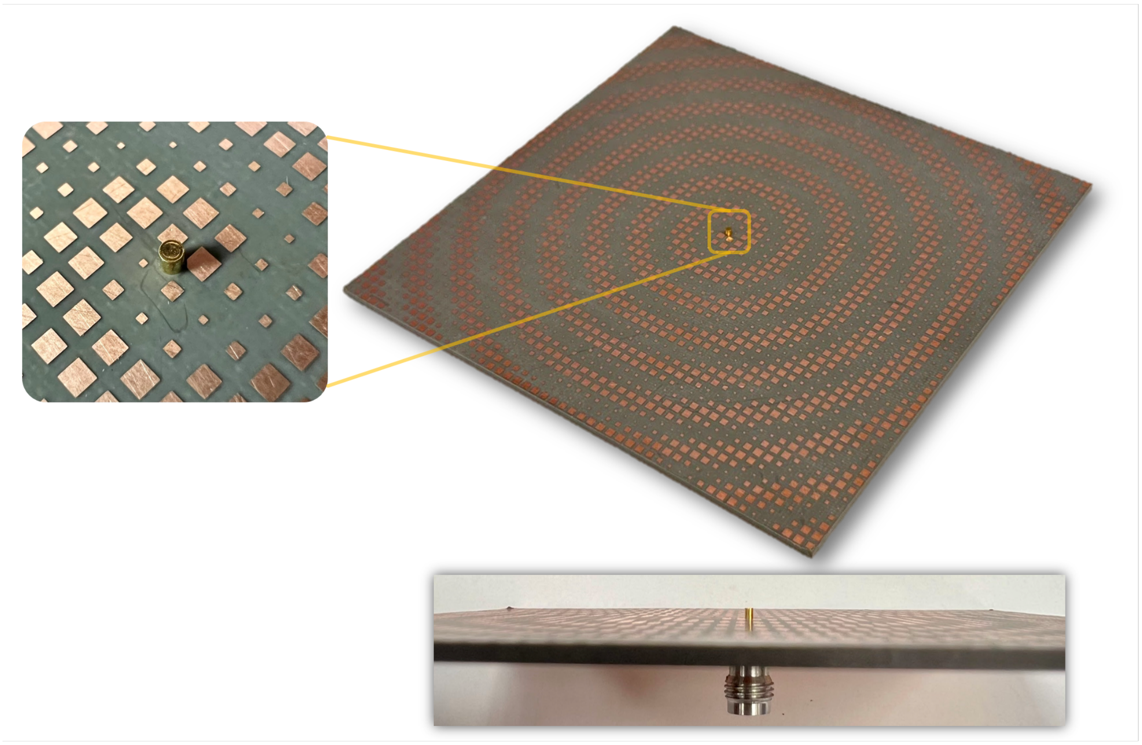

Figure S 6. Isometric and side view of our holographic metasurface antenna (HMA) prototype. In the inset, the monopole feed antenna is shown.

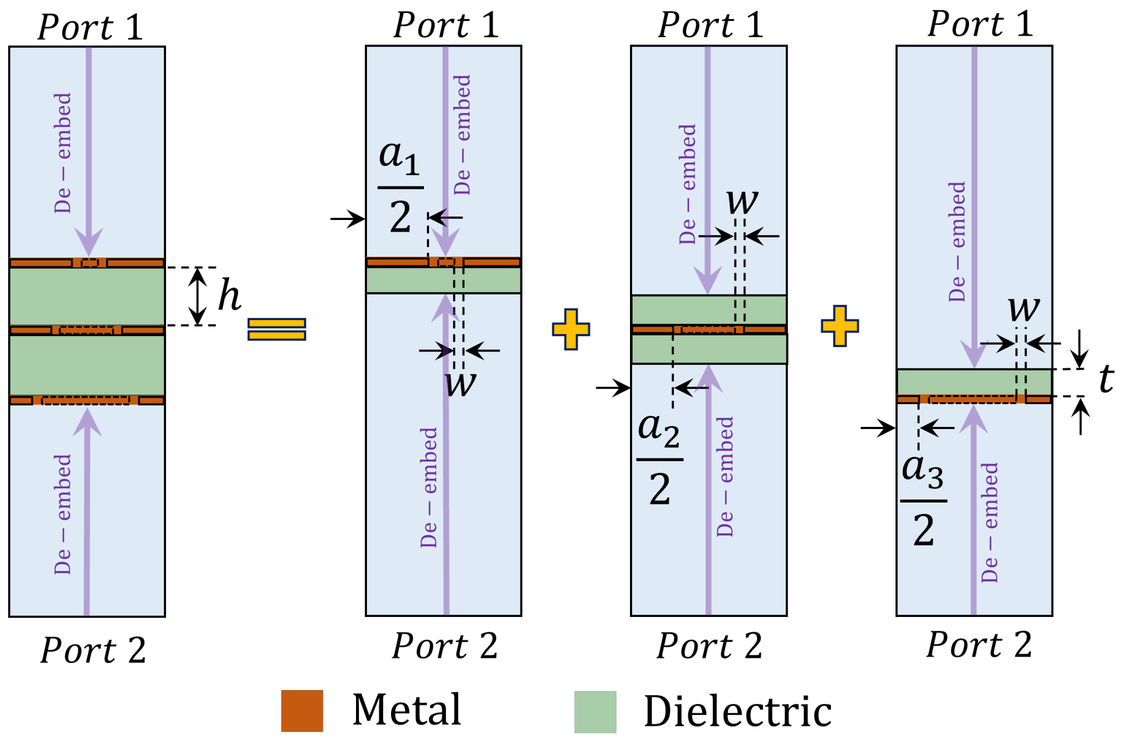

Figure S 7. A three-layer PSS unit-cell is split into three separate unit-cells (sections). To accurately evaluate the phase of the corresponding S-parameters we de-embed right above and below each layer.
